# Promoting Mental Health During the COVID-19 Pandemic: A Hybrid, Innovative Approach in Malaysia

**DOI:** 10.3389/fpubh.2021.747953

**Published:** 2021-10-06

**Authors:** Aimi Nadira Mat Ruzlin, Xin Wee Chen, Raudah Mohd Yunus, Ely Zarina Samsudin, Mohamad Ikhsan Selamat, Zaliha Ismail

**Affiliations:** Department of Public Health Medicine, Faculty of Medicine, Universiti Teknologi MARA (UiTM), Selangor, Malaysia

**Keywords:** health education, mental health, community health promotion, COVID-19, pandemic

## Abstract

**Background:** The COVID-19 pandemic has had monumental effects on the mental health of populations worldwide. Previous research indicated that programs and interventions using social networks can play a positive role in promoting mental health. Nevertheless, current evidence is largely derived from high-income regions, reflecting an urgent need for more studies in low- and middle-income settings.

**Objectives:** This paper aims to (a) describe the potential value of a hybrid health carnival in promoting mental health and increasing access to screening services; (b) assess the level of community engagement with the digital platform.

**Methods:** A mental health carnival was conducted with the theme of “Mind Your Mental Health” *(Cakna Kesihatan Mental)* in conjunction with the World Mental Health Day in Malaysia. This was a hybrid carnival that combined elements of face-to-face interactions and virtual learning. Free online therapy sessions were offered to high-risk groups identified during the screening process. Social media metrics were utilized to report the levels of community engagement and participants completed pre-and post-assessments to measure the program's impact on their knowledge.

**Results:** The carnival was attended by 515 participants (78.8% virtual participants). Social media metrics reported more than 5,585 reaches on Facebook for all the activities held throughout the event. Results from pre-and post-assessments showed significant improvement in the mean knowledge scores (*p* < 0.05).

**Conclusion:** This digital approach will continue to evolve by releasing new features and tools as a new frontier for high-risk populations and all individuals seeking mental health support and treatment.

## Introduction

Mental health is an integral and fundamental component of health, which can be defined as “a state of well-being in which every individual realizes his or her potential, can cope with the normal stresses of life, can work productively and fruitfully, and is able to make a contribution to her or his community” (1). The increasing magnitude and burden of mental health problems today are becoming a global public health concern. At the global level, depression affects more than 264 million people and is currently the leading cause of disability (2). From the total number of people living with this condition, nearly half reside in the Southeast Asian and Western Pacific regions (3). On the other hand, suicide accounts for 1.5% of all deaths worldwide, with more than 50% occurring in low- and middle-income countries (3).

Mental health promotion often refers to positive mental health, which is the desired outcome of mental health promotion interventions (4, 5). The fundamental aspects of mental health promotion include empowerment, competence, resilience, and supportive environments (6). Empowerment is a fundamental component of health promotion, and its main concept is to educate and enable individuals so they can have the knowledge (health literacy) and self-efficacy in maintaining good mental health and ensuring appropriate help-seeking behavior (7). Our program also provided learning opportunities to enhance personal competencies such as improving their coping strategies and develop emotional resilience which revolves around having a positive attitude toward mental health and mental health promotion as well as toward individuals (8). Supportive environments such as facilitating early access to professional psychological help and mental health services are actions that will also improve psychological well-being (9, 10).

Previous literature from social, biological, and neurological sciences highlighted the role of protective factors in the developmental pathways associated with poor mental health (4). Identifying and enhancing protective factors such as knowledge, literacy, beliefs and self-esteem, problem-solving and social skills are potential targets for mental health promotion with the explicit goal of promoting positive mental health and developing competence to promote well-being rather than preventing symptoms or the onset of disorders (11). The lack of protective factors may predispose a person to move from a mentally healthy condition to greater susceptibility to mental illnesses. According to a systematic review, mental health interventions also yielded significant and positive short-term and long-term effects on formal help-seeking behavior as well as mental health literacy and stigma especially if delivered to people with or at risk of mental health problems (12).

Mental health literacy refers to the ability to obtain and maintain positive mental health, understand mental health problems and their treatments, avoid stigma related to mental health problems, and enhance help-seeking efficacy (13). The low literacy and awareness could be attributed to many factors, among which are the stigma attached to mental disorders and the often perceived “intangible” nature of mental health. Empirical evidence emphasizes the importance of mental health awareness and literacy and shows that properly designed interventions can have tremendous and positive impacts. For example, an educational program to boost mental health awareness among university students in the Texas city of the United States demonstrated an increase in knowledge of, and reduction in stigma against, schizophrenia (14). Similarly, The Compass Strategy—a mental health literacy awareness campaign in Australia—was found to facilitate self-identified depression and awareness of suicide risk, improve the estimation of the prevalence of mental health problems, and reduce perceived barriers to help-seeking (15). Higher mental health awareness and literacy can empower people to act upon their knowledge and enable them to take charge of their well-being (16).

Given the rapid digital revolution in recent years, online mental health interventions have been increasingly used and recognized as effective in treating, preventing, and educating people with regards to mental health problems (17). A meta-analysis by Andrews et al. suggested that online interventions are comparable to traditional, face-to-face delivery of mental health treatments in terms of effectiveness (18). In addition, virtual mental health programs are said to offer various advantages including greater convenience (accessibility and flexibility), capacity to reach wider populations especially individuals who are socially isolated or are not able to be physically present, and provision of a cheaper and non-threatening avenue for psychological help (17, 19). Interestingly, the social networking sites (SNS) which are often associated with negative effects on mental health such as depression, loneliness, and self-esteem, are said to have opposite effects when used in a “healthy and mindful” manner, and with the right techniques (5, 20). For instance, Peek et al. corroborated the role of social media and blogging in mental health education and advocacy (21), while Kayrouz et al. highlighted the use of Facebook to engage with hard-to-reach populations for mental health programs and research (22).

Beyond mortality and morbidity, the impact of the COVID-19 pandemic across the globe has been unprecedented. The risks of transmissions and the counter-measures adopted have not only led to movement restrictions that resulted in economic shutdowns but caused a sharp rise in psychological distress, anxiety disorders, post-traumatic stress disorders, and other forms of negative feelings—such as excessive fear of death and paranoia—that could easily predispose individuals to mental illnesses (23–27). Due to the social distancing measures, forced closures of educational institutions, and reallocation of health resources to fight the pandemic, the demand for mental health literacy and services is unlikely to be met using conventional modes. In this context, digital or virtual platforms are the most practical alternative to compensate for the drawbacks of traditional mental health education and service delivery.

Despite the various proven benefits of online- and SNS-based mental health interventions, evidence on their use, level of engagement, and effectiveness in the context of low- and middle-income countries (LMIC) have been scarce. A systematic review on SNS-based mental health interventions for young people included nine studies in their final analysis, all of which were conducted in high-income countries (Australia, Hong Kong, and USA) (28). Similarly, another systematic review on online mental health interventions for depression among youths found 15 eligible studies, all of which were from high-income settings (29). This “geographical gap” was corroborated by Arjadi et al. in a 2015 review on online mental health interventions in LMICs, where the authors found only three studies; two from China and one from Romania (30). While we have witnessed a gradual burgeoning interest among LMIC academics and researchers in this field—especially with the advent of COVID-19—the body of evidence in this region remains low. Our study thus attempts to fill in this gap, and contribute to the existing knowledge on online-based mental health programs and interventions by providing a Malaysian perspective.

In this paper, we described a mental health program called the “Mind your Mental Health Carnival,” which was a hybrid form of mental health awareness campaign that combined face-to-face and online platforms, targeting mainly youths and university students: (a) describe the potential value of the hybrid health carnival approach in promoting positive mental health and increasing access to screening services; (b) assess the level of community engagement with the digital platform, and (c) evaluate the impact of the hybrid carnival on mental health literacy.

## Methods/Context

This study was a descriptive research which used quantitative methods to outline the potential benefits of the hybrid health carnival, to promote mental health in the midst of COVID-19 pandemic.

### “Mind Your Mental Health *(Cakna Kesihatan Mental)*” Hybrid Carnival

In conjunction with the World Mental Health Day, the “Mind Your Mental Health” Carnival was held from 9 to 11 October 2020. The project began with the development of educational materials as a strategy for mental health promotion and education. Posters were designed based on the Health Belief Model (HBM), a well-established theoretical approach that has been employed in various studies to explore the perception and belief of mental illness and mental health service utilization (31, 32). Examples of mental health issues and their corresponding HBM constructs, as well as health education messages to address these issues, are presented in Table 1. These messages were formulated by mental health experts and included the following principal constructs of HBM: (i) perceived susceptibility (what is mental health and who are at-risk); (ii) perceived severity (the seriousness of poor mental health effects); (iii) perceived benefits (of having good mental health), (iv) perceived barriers (to obtain good mental health and challenges in seeking social support), (v) perceived threat (of not having good mental health), and (vi) cues to action.

**Table 1:** Examples of mental health messages based on the HBM constructs.

**Constructs**	**Mental health issue related to constructs**	**Message examples for health educational materials to address construct issues**
Perceived susceptibility	Belief that one most likely will not be at risk of the health problem	End the stigma! Mental illness knows no boundaries and affects all walks of life adolescents, youth and adults.
Perceived severity	Belief that mental health is not a serious health problem	Mental Health is a “silent killer”! Mental health affects physical health and academic performance!
Perceived benefits	Belief that preventative action will be effective in reducing symptoms and improve mental health	With good mental health you can work productively and contribute to the society!
Perceived barriers	In denial that there is a problem and reluctant to talk to a counselor	It's OK to ask for help! Speak up for your needs and find support!

For perceived susceptibility, the public was informed regarding the risk factors of developing mental illness through various health education materials and provided screening by using the DASS-21 questionnaire (33). As for perceived severity, people are likely to engage in a given health-related behavior if they believe that the problem has serious consequences or will interfere with their daily functioning. Therefore, the varying levels of severity and symptoms of mental illness were elaborated to enable the audience to fully grasp the severity of certain mental health conditions. As for perceived benefits, we delivered insight on the benefits of good mental health. To overcome the barriers in accessibility, the availability of mental health services within the faculty and other related NGOs such as Befrienders was highlighted. Individuals with higher health consciousness have greater motivation to adopt health-seeking behaviors such as obtaining health-related information from the internet as the significant local information source. However, social media platforms provide access to unprecedented amounts of information and the use of social media as the main source has caused significant concerns given the reliability of this information. Therefore, the use of the HBM in creating educational health materials and incorporating scientific evidence from multiple perspectives is an effective tailored communication strategy in tackling current info-demics and unconventional health claims (34).

The carnival aimed to (i) create an accessible digital platform that provides learning materials for mental health awareness, and (ii) to provide appropriate screening tools for early identification of mental health issues and psychological support for high-risk groups.

### Advertisement of the Carnival

The target population for this event is adolescents, youths, and young adults who comprise the majority of SNS users in Malaysia. We chose this population because the onset of mental disorders peaks between adolescence and young adulthood (28). The 4th-year undergraduate medical students undergoing their public health medicine rotational posting were involved in preparing and hosting the health carnival as part of their curriculum. One month prior to the event, the students advertised the program by posting creative visual content on social media platforms and started customizing a countdown campaign to build momentum toward the event. Updated daily news and headlines were delivered automatically through the university's email system for faculty staff, undergraduate and graduate students. Given the role of social media as powerful marketing tools, the organizers used all successful media brands—Facebook, Instagram and Twitter—to promote the event. Diverse social media platforms would increase the opportunities to engage with more diverse groups of people in terms of age groups and preferences. Photography and short video competitions with the theme of “Mind Your Mental Health” *(Cakna Kesihatan Mental)* were opened to all Malaysian citizens. A poster describing a step-by-step neck and shoulder massage therapy was designed and posted on social media. This was followed by a video contest entitled “massage challenge” to encourage more users to learn the neck and shoulder massage techniques, to reduce muscular tensions, improve mood and relax the body.

To ensure that the event was a crowd-puller, the organizing team uploaded a total of 34 daily contents within the social media to increase public's awareness and educate the online community about mental illnesses such as anxiety disorders, hallucinations, depressions, panic disorders, obsessive-compulsive disorders and many more. Unique hashtags were adopted to gain likability and popularity on the trending list (i.e., #mindyourmentalhealth, #caknakesihatanmental, #mentalhealthawareness, #stopthestigma, and #loveyourself etc.).

### Activities of the “Mind Your Mental Health” Hybrid Carnival

Upon arrival at the carnival site, participants were required to comply with Standard Operating Procedures (SOPs) related to COVID-19, including registering themselves through the *My Sejahtera* application developed by the Government of Malaysia, checking their body temperature, and maintaining physical distancing of one-meter. The program of the events included the following:

(i) The hybrid carnival began with an opening ceremony, which took place within the faculty and was broadcasted live through Facebook and Instagram.
(ii) Subsequently, a “Chairobic” session was conducted which featured a simple set of gentle exercises performed while seated on a chair for 20 min. This activity was aimed to educate the public on regular exercises at home and/or at work, as a way which was essential to inculcate a healthy lifestyle through exercise as one of the measures to reduce stress and relax the mind. The steps were simple and included fist-clenching, arm-stretching, seated side bend stretching, and leg raising.
(iii) The program continued with a motivational forum entitled “Busting the Myth” featuring a mental health professional and a Muslim scholar, *Ustaz*, to expose the audience to a more holistic perspective of mental health. The question-and-answer sessions were opened to both on-site and virtual attendants.
(iv) “Posteria” corner exhibited educational materials, which were developed based on the HBM. These posters were displayed in the faculty and posted digitally on social media. Facebook live streaming video sessions were held to further elaborate on mental health education.
(v) Online mental health trivia quizzes were created using Instagram Story to raise public awareness on mental health issues and to fully grasp the topic of mental health.
(vi) Two short videos were uploaded to social media. The first video demonstrated a step-by-step guide on deep and rhythmic breathing techniques to reduce symptoms of anxiety disorders such as general anxiety, social anxiety, and panic attacks. The second video was a learning tool that demonstrated simple neck and shoulder massage therapy as a form of stress relief.
(vii) Counseling and mental health services were made available to assist those in need of mental health support. Participants were encouraged to have their mental health screened and/or meet up with the professional counselors for assistance. The screening was done among 88 respondents using the DASS-21 questionnaire to assess symptoms of depression, anxiety, and stress.
(viii) The closing ceremony was broadcasted to the virtual audience with an estimated 1,400 views on multiple social media platforms. A closing speech was delivered by the university's Deputy Dean of Academic and Student Affairs, highlighting the importance of raising awareness on mental health issues, fighting stigma related to mental illnesses, and seeking help early.

### Research Tools

Participants were encouraged to have their mental health screened and/or meet up with the professional counselors for assistance. Mental health screening was performed using the Depression, Anxiety, and Stress Scale (DASS-21) (33). The depression scale in DASS-21 assesses a range of depressive syndromes including dysphoria, hopelessness, and lack of interest/involvement. A higher score indicates a higher level of depression. The anxiety scale assesses the subjective experience of the anxiety effect, autonomic arousal, skeletal muscle effects, and situational anxiety. The stress scale assesses difficulty relaxing, being irritable/- overreactive and impatient, and being easily upset/agitated. The Malay version of DASS-21 was used, as its reliability (Cronbach's alpha = 0.95) and construct validity have been well-established (33).

Apart from that, the pre-and-post self-assessment forms were distributed during the event to detect any change in mental health awareness among the participants. Mental health knowledge was measured using the Mental Health Knowledge Questionnaire (MHKQ) (35).

### Analysis

This social media-based intervention which was promoted using multiple SNSs such as Facebook, Instagram, and Twitter included evaluation metrics that were commonly reported for digital campaigns. Relevant key performance indicators (KPIs) such as measures of campaign awareness and proximal impact measures of engagement (i.e., responses) were adopted to reflect and measure the effectiveness and performance of the campaigns. Social media metrics measuring campaign awareness were used to quantify the total number of reaches that represent the number of people that have viewed the campaign by social media. Proximal impact measures of engagement represent engagement on social media and are comprised of the total number of likes, shares, followers, or comments on social platforms (36). Therefore, there were two main measures of social media KPIs that were evaluated in this hybrid health carnival program:

a) Awareness social media metrics (e.g., reach: number of viewers that a unique post has reached)
b) Engagement social media metrics (e.g., likes, shares, followers, and comments)

Data obtained from the DASS-21 Questionnaire was exported to SPSS version 23 for analysis. Respondents with a depression score of 10 and more were considered to have depression, those with an anxiety score of eight and more were considered to have anxiety, and those with a stress score of 15 and more were classified as having stress (33). Pre-and-post assessment scores were recorded and summarized by calculating the means and standard deviation (SD). Paired *t*-tests were used to compare the means of the two samples. Statistical significance was set a priori at *p* < 0.05.

### Ethical Considerations

Participants attending the carnival were informed about the voluntary nature of having their mental health screened and their information would be kept confidential. The form did not contain personal identifiers, and participants were given a choice of whether they would like to be referred to a professional counselor if they were found to be at-risk. Aggregate data analysis was performed to protect respondents' anonymity and confidentiality. This project was granted ethical approval from the ethical review board of the institute where the carnival was held.

## Results

The hybrid carnival had a total of 515 participants, whereby 78.8% participated virtually. The event recruited 1,104 followers through Instagram, 370 likes through Facebook, and 88 followers on Twitter. All the available online campaigns used different modes of health promotion and educational interventions. According to the objectives of the campaigns, different content-based communication strategies were adopted.

Most participants obtained information regarding the event through online communication in social media (43.2%), followed by WhatsApp (29.79%), and peers (25.2%). To summarize the social media metrics for all the daily contents during the preparation, Instagram metrics tracked a total of 1,150 likes and 15 comments, Facebook metrics tracked a total of 5,236 reaches, 401 likes, and 116 shares, while Twitter metrics tracked 160 retweet and 158 likes. As per social media metrics traced for a single post, it was revealed that the number of reaches in Facebook ranged from 38 to 704 in a single post and up to 457 views on Instagram. The virtual countdown posts had a high number of reaches and engagement with a total of 901 on Instagram and Facebook.

The two health educational videos received good response rates. The first video demonstrating breathing techniques successfully achieved 457 views, 101 likes, and 29 comments on Instagram; 489 reaches, 188 views, and 15 likes on Facebook; 137 views, 12 likes, and 12 retweets on Twitter. Meanwhile, the second educational video on massage therapy had 188 plays on Facebook; and 261 views, 66 likes, and three comments on Instagram.

Participants were asked to complete a baseline self-assessment of their mental health knowledge before and after the 2-day event. Overall, results revealed a significant improvement and performance in the mean knowledge scores (*p* = 0.001), whereby the mean paired difference was 2.56 (1.90, 3.23). The mean (SD) knowledge scores post-carnival, reported at 18.27 (1.761), was significantly higher than the mean (SD) knowledge scores pre-carnival that stood at 15.70 (3.407).

Table 2 shows the social media metrics KPIs for each of the activities held during the hybrid health carnival. Results indicate a high combination of reach and engagement metrics for both social media platforms with high follower growth in the health carnival's Instagram page (1,104 followers). Overall, Facebook had the highest engagement reach of all the activities held during the carnival.

**Table 2:** Social media metrics of each activity conducted in hybrid health carnival.

**Activities**	**Facebook**	**Instagram**
Live session presenting the poster	342 people reached 155 plays	215 views 36 likes
“Chairobic” session	918 people reached 345 views 36 likes 31 comments 21 shares	564 views 50 likes 15 comments
Forum “Busting the myths”	3,488 people reached 1,219 views 88 likes 70 comments 30 shares	902 views 88 likes 20 comments
Closing ceremony	837 people reached 368 plays	5,000 likes 1,000 views 95 comments

**Figure 1:**
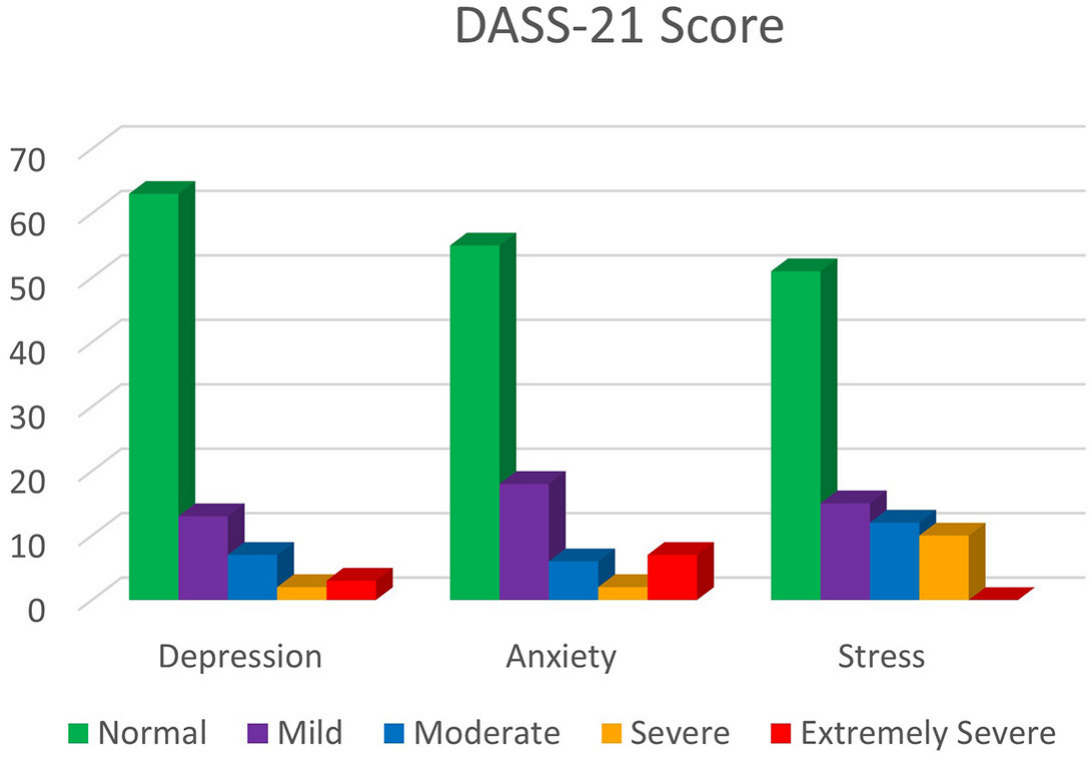
Bar Graph of DASS-21 scores according to severity among participants attending mental health screening.

From the mental health screening based on DASS-21, the prevalence of depression was 25%, anxiety was 33%, and stress was 37%. These figures are illustrated in Figure 1. Those who had severe or extremely severe levels of depression, anxiety, and/or stress (a total of 16%) were categorized as high-risk groups and referred to a licensed professional counselor for a counseling session.

## Discussion

The overarching aim of this study was to describe the potential value of a hybrid health carnival approach in promoting positive mental health and increasing access to mental health screening services for the community. Based on the sizable number of participants, social media metrics, screening uptake, and pre- and post-assessment of mental health knowledge, a hybrid health carnival approach to mental health promotion is demonstrably and evidently an effective modality to engage communities and increase mental health literacy.

The hybrid health carnival, which was grounded by the principles of mental health promotion (6), used a blended approach to health promotion and education delivery, by utilizing physical-based and web-based platforms to deliver mental health content. Traditional face-to-face interventions continue to be successful in health promotion initiatives (37), and thus all components of the hybrid health carnival were delivered to participants attending the carnival on-site. Concurrently, social media was adopted as a tool to deliver mental health promotion materials *via* web-based platforms. The use of social media as a tool to deliver health interventions is fitting in this day and age where the nature of interpersonal interactions has evolved in line with advancing digital technology. Social media platforms are progressively developing as a rich source of mass communication as they offer easy, cost-effective access to a large number of people across geographic distances in a short time frame (38). Furthermore, interventions using social media platforms address some of the limitations observed by traditional health communication strategies by increasing the potential for interaction, customization, and participation (39).

Indeed, widespread public engagement with social media creates a ready platform for its application in the health field (40) and health promotion professionals have begun to recognize the potential of social media for enabling and empowering consumers in health and healthcare-related interactions (41). Previous studies have shown that the use of mobile technology and social media in delivering health promotion interventions produces successful outcomes. Various mobile phone interventions to address various health concerns such as reducing obesity, increasing physical activity, and reducing HIV risk behaviors have demonstrated measurable beneficial impacts on participants (42, 43). Similarly, interventions using social networking sites were reported to be effective in promoting changes in health-related behaviors (44).

In the hybrid health carnival, various communication strategies including social media were employed to create awareness and gain the interest of the target audience. Social media metrics were used to gauge participants' interest and acceptance of the hybrid health carnival, as they provide valuable information about reach and usability (40). As shown in this study, by utilizing popular social media platforms including Facebook, Instagram, and Twitter to publicize the hybrid health carnival, the reach for the hybrid health carnival was enhanced. This was evident from our findings, where the majority of virtual carnival participants (43.2%) had become aware of the hybrid health carnival *via* social media platforms. Indeed, according to event statistics, 76% of event organizers use Twitter to promote their events, and 88% of companies use social media to create awareness before an event, the most popular being Facebook, Twitter, and Instagram (45). Besides social media, several other modalities were utilized to promote the hybrid health carnival, including flyers, daily news *via* university email, WhatsApp, and word-of-mouth communication. This multimodal approach is the most effective method to reach audiences about health issues (46).

In addition, as part of the promotion strategy, the organizers of the hybrid health carnival provided daily news on the event *via* university email and social media platforms as well as conducting a countdown campaign, which helped generate momentum toward the event and kept it in the spotlight for longer. This is in line with the marketing tactics advocated by marketing strategists to help build excitement, sustain interest, and get some last-minute sign-ups from the target audience (45). Similarly, unique hashtags were also used to gain likability and popularity. As a result, the organizers were able to pull quite a number of crowds to attend the virtual session and the live event physically, despite the short period of time allocated to conduct the hybrid health carnival.

In terms of community engagement, live broadcasting *via* Facebook and Instagram was able to generate a respectable level of engagement among participants. These platforms allow not only a wider reach, but also improve participants' engagement as followers are able to ask questions and have them answered in real-time (45). As in previous studies, interactive communication is more effective than linear, i.e., one-way, communication (39), and multidimensional interventions and participant interactivity are pivotal toward successfully reaching diverse audiences (47). Moreover, the interactive and participative nature of content delivery such as the short video contest and photography competition which combined interpersonal and mass media communication has been argued to be more impactful on health behavior (48). On the whole, the social media metrics for each of the activities conducted and the numbers of participants being screened and referred for counseling suggest that a fair level of engagement from the attendees was achieved throughout the carnival.

One of the outcomes assessed during the hybrid carnival was mental health literacy, in keeping with the notion of promoting positive mental health by empowering communities (6). Improved mental health literacy was observed among study participants as evidenced by the significant increase in mental health knowledge among participants who attended the hybrid health carnival (*p* = 0.001). This is highly encouraging given that mental health literacy is fundamental toward empowering whole communities to take action for better mental health (16). Moreover, mental health literacy curbs the current phenomenon of infodemic and misinformation pertaining to mental health that may be associated with it as more and more people become reliant on social media as a means to keep up to date with current information (49).

Other carnival components aimed at improving communities' competence and resiliency included the health educational videos demonstrating breathing techniques and massage therapy. The ultimate aim of these videos was to improve one's coping skills and ability to cope with mental distress, thereby increasing the options available to individuals to exercise more control over their health and their environment to make choices conducive to health (50). Though the efficacy of these videos on enhancing personal skills was not assessed, the social media metrics suggested that participants received them favorably and may be more inclined to practice the stress-reducing techniques in daily life. Finally, the hybrid health carnival also aimed to enable supportive environments, in keeping with the principles of mental health promotion. Though this was again not assessed directly, the screening uptake and subsequent referrals to counselors for those at-risk suggests that participants were comfortable enough to undergo screening despite the stigmatization of mental health disorders. The creation of supportive environments is imperative to the success of any mental health promotion interventions, as mental health is mediated by the interaction between the individual, the environment, and wider social forces, underscoring the need for mediating structures such as home, schools, workplaces and community settings to be accommodative (50).

In relation to evidence pertaining to the value of hybrid health carnivals within the local context, at present, there is scarce evidence to support this modality in delivering mental health promotion interventions within the Malaysian setting. However, studies published elsewhere have indicated encouraging results from health promotion campaigns using social media platforms for mental health advocacy. In India, a mental health promotion campaign on suicide prevention, tobacco cessation, and migraine reported satisfactory reach and level of engagement from social media users (38). Similarly, a social media intervention conducted in Canada to raise awareness and improve attitudes toward mental health issues among youths and young adults demonstrated increased mental health literacy outcomes (51). With regards to treatment, a systematic review explored the effectiveness of blended interventions for the treatment of mental health disorders and reported that blended therapy may save clinician time, lead to lower dropout rates, and help maintain initially achieved changes within psychotherapy in the long-term effects of inpatient therapy (52).

To give further credence to the potential of hybrid health carnivals in delivering mental health promotion initiatives, comparisons between online interventions and conventional interventions have shown that online interventions are generally at least as effective as traditional face-to-face interventions (37). Thus, a blended intervention combining face-to-face and internet-based approaches for addressing health issues is appropriate, especially in the COVID-19 pandemic era where social distancing is the norm. As both face-to-face and online interventions have their respective advantages and disadvantages and functions, this form of intervention may be the best method of facilitating health promotion initiatives, as suggested by previous studies (37). In the post-COVID-19 era, this is especially pertinent given the psychological distress associated with the COVID-19 pandemic as well as the need to accommodate COVID-19 control measures.

There are some limitations to this study. Recruitment of study participants was conducted *via* university emails and advertisements on social media platforms, which may have limited participant demographics. This is because some segments of the community especially youths from low socioeconomic or marginalized backgrounds may have been excluded, thus our study participants are not representative of the overall population. Another limitation stems from the use of social media metrics to gauge the response to the hybrid health carnival. These indices may not have reflected the true impact of the carnival, as the application of metrics to track and evaluate social media is still in its infancy, and there is currently limited understanding on how to measure social media impacts most effectively (40). Furthermore, the study was not able to determine whether the meaningful engagement was achieved from the use of social media, as the metrics do not indicate whether participants were “just stopping by” or actually engaging with the content as intended (40). In addition, the approach to measure this carnival's impact lacked a qualitative perspective (e.g., verbal, or written feedback from students or audience), which could have been more relevant to assess “meaningful engagement.” With regards to the change in scores said to indicate the impact of the program on mental health literacy, post-event measurement was conducted once. Whether or not such an immediate impact (increase in mental health literacy) is sustainable and prolonged cannot be determined.

## Conclusions

The hybrid mental health carnival using social media is a new approach for delivering engaging health messages and promoting mental health in Malaysia. Despite the multiple limitations of virtual communications and online platforms, the current digital transformations occurring at the global level—accelerated by the COVID-19 pandemic—provide numerous “windows of opportunities” for innovation and creativity to flourish with regards to health intervention deliveries. The role of digital platforms in the health field is increasingly recognized, therefore health care providers and policymakers have no choice but to keep up with these changes and make the most out of them. For future research, it is recommended that a more robust method is designed to measure the impact or effectiveness of similar virtual health campaigns.

## Data Availability Statement

The datasets presented in this article are not readily available because dataset request is subjected to approval by the Research Ethics Committee. Requests to access the datasets should be directed to pjimedic@uitm.edu.my.

## Ethics Statement

The studies involving human participants were reviewed and approved by Universiti Teknologi MARA (UiTM) Research Ethics Committee [REC/05/2020(MR/101)]. The patients/participants provided their written informed consent to participate in this study.

## Author Contributions

All authors contributed to the conceptualization and writing of this manuscript.

## Funding

This health carnival event was funded by the Universiti Teknologi MARA Faculty of Medicine. Funder of the event is not involved in the manuscript review.

## Conflict of Interest

The authors declare that the research was conducted in the absence of any commercial or financial relationships that could be construed as a potential conflict of interest.

## Publisher's Note

All claims expressed in this article are solely those of the authors and do not necessarily represent those of their affiliated organizations, or those of the publisher, the editors and the reviewers. Any product that may be evaluated in this article, or claim that may be made by its manufacturer, is not guaranteed or endorsed by the publisher.
