# Supplementary material for: Promoting Mental Health During the COVID-19 Pandemic: A Hybrid, Innovative Approach in Malaysia
Source: Front Public Health. 2021 Oct 6;9:747953. doi: 10.3389/fpubh.2021.747953 (PMC8526842; doi:10.3389/fpubh.2021.747953)
Supplement: Supplementary file 1 [file Table_1.DOCX]

Supplementary Material

# Multimedia Appendix

Video: <https://spark.adobe.com/video/aWCm6OjYp8Fsw>

Digital story telling: <http://bit.ly/caknakesihatanmental>
